# Evaluation of High Dynamic Range Imaging Methods for Luminance Measurements

**DOI:** 10.3390/jimaging12030114

**Published:** 2026-03-09

**Authors:** Lou Gevaux, Alejandro Ferrero, Alice Dupiau, Ángela Sáez, Markos Antonopoulos, Constantinos Bouroussis

**Affiliations:** 1 Laboratoire Commun de Métrologie LNE-Cnam (EA 2367), Conservatoire National des Arts et Métiers, 93210 Saint Denis, France; 2 Instituto de Óptica “Daza de Valdés”, Consejo Superior de Investigaciones Científicas, 28006 Madrid, Spain; alejandro.ferrero@csic.es (A.F.); angela.saez@csic.es (Á.S.); 3 Institute of Communication and Computer Systems, 15773 Athens, Greece; 4 Eidgenössisches Institut für Metrologie METAS, 3003 Bern, Switzerland

**Keywords:** HDR, luminance, imaging, camera model, metrology

## Abstract

Imaging luminance measurement is increasingly used in lighting applications, but the limited dynamic range of camera sensors requires using high dynamic range (HDR) imaging methods for evaluating scenes with large luminance contrasts. This work aims at investigating how parameters of HDR imaging techniques may impact luminance measurement accuracy, using a numerical evaluation. A numerical simulation framework based on a digital twin of an imaging system and synthetic high-contrast luminance scenes is used to introduce controlled systematic error sources and quantify their impact on HDR luminance accuracy. The results support the identification of HDR approaches most suitable for producing luminance measurements traceable to the International System of Units (SI).

## 1. Introduction

Imaging instruments are increasingly used to measure luminance for lighting applications. They allow the luminance of an entire scene to be captured much more quickly than with point measurement instruments, which evaluate only one position per acquisition. They also enable dense measurements, providing luminance values for each pixel of the scene with high spatial resolution. As a result, complex scenes that were previously difficult to study can now be photometrically characterized more efficiently. However, the dynamic range of matrix sensors used in imaging instruments (CCD or CMOS, typically) is generally lower than the range required for the scenes to be studied, particularly when investigating issues such as discomfort, glare, or obtrusive light, where both very bright sources and dark backgrounds must be accurately measured. To overcome this equipment limitation, high dynamic range (HDR) imaging techniques [1,2] are employed [3,4,5]. These methods were developed many decades ago and have been a topic of high interest since the democratization of digital imaging in the 1990s. Today, HDR techniques are implemented in a large number of consumer devices, either through multi-exposure fusion or single-exposure architectures (e.g., dual-gain or multi-conversion sensors), increasingly enhanced by AI methods, notably in smartphones. Nevertheless, the performance sought in recent developments is often focused on producing visually pleasing images, which differs from the needs of the lighting applications we are interested in. Indeed, our objective is to obtain the best possible estimate of luminance values with associated uncertainties in a way that is traceable to the International System (SI) of units.

In this context, the HDR imaging methods currently used for luminance measurement rely on well-established approaches, consisting of combining several low dynamic range (LDR) images acquired at different exposure levels [6,7,8]. An LDR image is a set of digital values coded by the number of bits corresponding to the dynamic range of the sensor. In a very simplified way, we can say that, for a linear camera, each value associated with a pixel of the sensor is proportional to the luminance of the portion of the scene imaged on the pixel, to the sensitivity of the camera, and to the integration time. This is true if the amount of light reaching the pixel is neither too low, in which case the signal is lost to the noise, nor too high, in which case the pixel becomes saturated. Luminance measurement is therefore possible over a limited range of values using a single LDR image, the lower and upper bounds of which can be shifted by adjusting the camera’s sensitivity (sensor gain, lens aperture, addition of neutral densities, etc.) or the integration time. Consequently, to evaluate scenes containing a very wide range of luminance values, it is necessary to take several images at several exposure levels and combine them using HDR merging algorithms. The main purpose of these algorithms is to produce images with the largest possible dynamic range while ensuring that the signal-to-noise ratio (SNR) of the luminance estimate for each pixel remains sufficiently high.

The quality of HDR measurements is typically affected by factors related to the measuring instrument, the LDR image acquisition strategy, the choice of HDR merging algorithm, and its parameter settings. The question of the best HDR imaging method, including the best merging algorithm, has been addressed before [9,10,11,12,13]. However, to our knowledge, most studies focused on the effects of sensor noise only and did not consider other significant effects, which might have a significant impact on the calculated HDR values. In this study, we evaluate a set of relevant parameters, including sensor non-linearity, inaccuracies in integration time, algorithm threshold selection, and temporal stability of the scene. Our objective is to assess the performance of HDR imaging methods in producing accurate and traceable luminance measurements, considering the aspects relevant to lighting applications. To this end, we analyze, through a numerical approach, the impact of different HDR-method parameters on luminance measurement accuracy. This approach relies on a camera “digital twin” implemented in MATLAB software (R2023b), which models the acquisition of an image by an imperfect digital camera. The camera digital twin is used to replicate the HDR imaging process applied to capture a virtual scene, yielding a synthetic HDR luminance image that can be compared to the ground truth scene. This approach allows us to study sensitivities for each parameter and to determine whether one type of HDR merging algorithm performs better than the others or not. In this study, we chose to focus on parameters whose impact may depend on the type of HDR merging algorithm applied, as well as on parameters that can be directly adjusted by the camera user. In this context, luminance inaccuracies caused by vignetting and stray light generated by the objective lens were not considered. The latter, which is often the most limiting factor for accurate imaging measurements, will be addressed in a separate study, as experimental evaluations are more appropriate than the digital approach presented here. The HDR imaging methods are introduced in Section 2, where the relevant parameters to be tested are also presented. The impact of noise for different HDR imaging methods is theoretically analyzed in Section 3. The conclusion from this analysis is the basis for selecting candidate methods to be further explored in more realistic conditions, where multiple error sources beyond photon noise are considered. This exploration is detailed in Section 4 and Section 5. Section 4 describes the evaluation method, in which camera digital twins are used to synthetically generate HDR images affected by various sources of error, allowing the evaluation of their impact on the preselected HDR imaging methods. The results obtained using this evaluation method are presented in Section 5, followed by the conclusions in Section 6.

## 2. HDR Imaging Methods and Parameters

Measuring luminance in scenes with a wide dynamic range is achieved using HDR imaging methods that rely on capturing a series of low dynamic range (LDR) images at varying exposure levels and combining them into a single image using an HDR merging algorithm. The HDR imaging methods evaluated in this study comprise the camera properties, the acquisition parameters of the LDR images, the HDR merging algorithm used, and its associated parameters. These aspects of the method, some of which are tested in our evaluation, have been detailed in [14] and are summarized in this section.

### 2.1. Image Acquisition

#### 2.1.1. Camera Properties

For an ideal camera, each pixel value *D*, expressed in count (or digital number), is proportional to the luminance *L* of the scene area imaged on the pixel, the camera luminous responsivity *s*_V_, and the integration time *t*. In this study, we only consider linear cameras, for which no camera response function (CRF) is applied in the image acquisition process. The ideal image acquisition by a linear camera can be expressed as:

(1) $$D=s_{V}\cdot t\cdot L\;.$$

The luminous responsivity *s*_V_ depends on parameters adjustable by the user (e.g., sensor gain and objective aperture) as well as on intrinsic camera parameters, such as the quantum efficiency of the sensor or the transmittance of the objective lens. This conversion is spectral, but for simplicity, we consider here that the camera is perfectly adjusted for luminance measurement, for example, by using a filter yielding a spectral responsivity that perfectly matches the spectral luminous efficiency function V(λ) [15].

**Figure 1:**
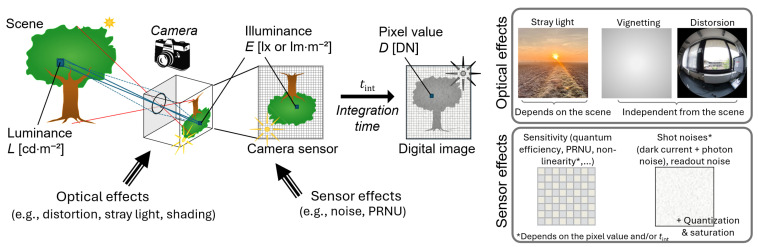
Schematic representation of the digital image capture process (reproduced from [16]).

For a real camera, image quality is degraded by several effects arising from the objective lens and the digital sensor, as illustrated in Figure 1 [16,17]. The objective lens impacts the image through effects including optical aberrations, geometric distortions, shading, and stray light caused by light scattering in the objective lens [18]. When the resulting imperfect optical image is captured by the matrix sensor, additional sources of distortion and noise arise: pixel response can vary between pixels (described by photo response non-uniformity–PRNU), and the signal is impacted by several types of noise (photon noise, thermal noise, and readout noise). Additionally, artifacts such as blooming (in CCD sensors) and smearing can occur when capturing intense light sources. Finally, the quantization of the signal into a count value encoded into a limited number of bits introduces some quantization noise, and the pixel value is clipped to the maximum possible value. Many of these effects can be modeled using mathematical equations and parameters that depend on the camera properties. The expression in the Equation (1) can therefore be rewritten to represent a real-world image acquisition process, as proposed in Section 3.

Some of the effects listed above depend on the scene and acquisition parameters, but some effects introduce systematic errors independently of the signal value and integration time. For the latter, their impact on HDR images is independent of the type of HDR merging algorithm applied. These effects, which include the optical effects (e.g., stray light and shading) and PRNU, are therefore not considered in our evaluation. The camera properties included in the study are the camera readout noise, rate of thermal noise, photon noise (related to the gain), sensor bit-depth, and non-linearity.

#### 2.1.2. Exposure Variation Methods for HDR Imaging

Several techniques can be used to capture a series of LDR images at different exposure levels. These include adjusting the integration time or shutter speed, sensor gain (ISO), and aperture (F-stop—though this should be avoided to maintain stable vignetting and depth of field); using neutral density (ND) filters; summing an increasing number of repeated captures; or combining these approaches [1,14]. The choice of technique depends on the constraints imposed by the application, the properties of the scene (e.g., dynamic range, temporal stability, light modulation), as well as the operational limits of the camera. In this study, we consider only LDR image series acquired with varying integration time, a method that is generally simple to implement and that provides good repeatability. However, depending on the technology of the camera shutter (e.g., for mechanical shutters), errors in the integration time values, especially for short integration times, can occur. The impact of these errors on the reconstructed HDR luminance image is considered in the numerical evaluation conducted in our study.

#### 2.1.3. Acquisition Parameters

The parameters of the LDR image series acquisition are the number of captured images and the integration time associated with each image in the case where integration time variation is used. It is common practice to define minimum and maximum integration times and to distribute the integration times of the acquired images between these two limits following a logarithmic progression, for instance by doubling or quadrupling the integration time between successive acquisitions.

The minimum integration time is constrained by the camera’s shortest integration time and by the scene characteristics. If there is temporal light modulation in the measured scene, the shortest integration time should be longer than the modulation period, ideally a multiple of the modulation period [19]. The minimum integration time is chosen to maximize the pixel value of the brightest scene area without saturating.

The maximum integration time is constrained by the camera’s characteristics and by the temporal stability of the scene, as objects might move during the LDR image series capture. Long integration times will allow for capturing areas with low luminance levels; however, thermal noise in the sensor, which increases with integration time, might strongly degrade the SNR of the measurement.

Finally, the integration time ratio between two LDR images of the series, directly related to the number of captured LDR images, is mainly limited by the temporal stability of the scene and the maximum acceptable acquisition time for the user. We refer to this parameter as the integration time reduction factor.

### 2.2. HDR Merging Algorithms

Once the LDR series is acquired, images are combined using an HDR merging algorithm, which defines whether a pixel is considered “well-exposed” or not, and how the HDR image value is computed when a pixel is well-exposed across several images of the LDR series. In this study, three types of algorithms have been tested: the “best exposure” algorithm, the “weighted average” algorithm (in the linear and logarithmic domains), and the “linear regression” algorithm [2,12]. These algorithms have been preselected for their simplicity (case of the best exposure algorithm), their potential to reduce noise (case of the weighted average algorithm), and their possible robustness to dark offsets (case of the linear regression algorithm). No AI-based HDR approach has been considered in this study. Although such methods may perform well in dynamic scenes and for perceptual quality optimization, they generally lack physical interpretability and uncertainty traceability, which limits their suitability for metrological luminance measurements.

In the following models, which give the HDR luminance value for a single pixel, *s*_V_ represents the luminous responsivity of the camera, *D_i_* is the pixel value on the *i*th image of the LDR series of *M* images, *D*_0,*i*_ is the associated dark signal, and *t_i_* is the integration time for the *i*th image. The minimum and maximum pixel values, *D*_min_ and *D*_max_, are the algorithm parameters used to define well-exposed pixels.

The best-exposure algorithm, expressed by Equation (2), constructs the HDR image by selecting the best-exposed pixel from the LDR images, which is the pixel with the highest value below the maximum usable value:

(2) $$L_{HDR}=\frac{1}{s_{V}}\cdot \frac{D_{i}-D_{0,i}}{t_{i}},\;t_{i}=\max\left\{t_{i}\;such\;that\;D_{min}\leq D_{i}\leq D_{max}\right\}.$$

The weighted average algorithm aims at reducing measurement error by using information from all the well-exposed pixels across the LDR images. For this, the pixel values are averaged, applying a weight factor that can depend on parameters such as the pixel value, SNR, or integration time. This algorithm can be expressed in the linear domain (Equation (3)) and in the logarithmic domain (Equation (4)):

(3) $$L_{HDR}=\frac{1}{s_{V}}\cdot \frac{\sum_{i=1}^{M}q_{i}w_{i}\frac{D_{i}-D_{0,i}}{t_{i}}}{\sum_{i=1}^{M}q_{i}w_{i}}\;,$$

(4) $$L_{HDR}=10^{\left(\frac{\sum_{i=1}^{M}q_{i}w_{i}\left(\log_{10}\left(\frac{D_{i}-D_{0,i}}{s_{V}}\right)-{log}_{10}\left(t_{i}\right)\right)\;}{\sum_{i=1}^{M}q_{i}w_{i}}\right)},$$

with

(5) $$q_{i}=\left\{\;\begin{array}{cc} 1 & \left(D_{min}\leq D_{i}\leq D_{max}\right)\\ 0 & otherwise \end{array}\right.\;.$$

Finally, the linear regression algorithm uses the linear relationship between the pixel value and the integration time to estimate the luminance value associated with the pixel from its well-exposed values across the LDR series, based on a least-squares regression, expressed in Equation (6). For this equation, *q_i_* is also defined according to Equation (5):

(6) $$L_{HDR}=\frac{1}{s_{V}}\cdot \frac{\sum_{i=1}^{M}q_{i}\cdot \sum_{i=1}^{M}q_{i}t_{i}(D_{i}-D_{0,i})-\sum_{i=1}^{M}q_{i}t_{i}\cdot \sum_{i=1}^{M}q_{i}(D_{i}-D_{0,i})}{\sum_{i=1}^{M}q_{i}\cdot \sum_{i=1}^{M}q_{i}t_{i}^{2}-{\left(\sum_{i=1}^{M}q_{i}t_{i}\right)}^{2}}\;.$$

The threshold values *D*_min_ and *D*_max_ define the range set to limit the impact of noise at small values and non-linearity and saturation near the sensor’s upper limits.

## 3. Theoretical Impact of the Noise on the HDR Imaging Methods

Among the HDR merging algorithms presented above, the weighted average algorithm has been developed to reduce noise in HDR luminance measurements. However, the performance of the algorithm, which can be evaluated as its ability to reduce noise, strongly depends on the choice of weight scheme. We propose in this section a theoretical study to better understand how the weighting factor impacts the results, under the assumption that the main contribution to noise is photon noise (also called shot noise), where the variance of photoelectrons equals its average. In this case, the variance of the signal at a given pixel under a given exposition is proportional to the integration time.

For simplicity, we set the luminous sensitivity *s*_V_ to 1 in this theoretical study. Similarly to Equation (3), the general weighted-average HDR method can be expressed as:

(7) $$L_{w}=\frac{\sum_{i=1}^{M}w_{i}L_{i}}{\sum_{i=1}^{M}w_{i}}=\frac{\sum_{i=1}^{M}w_{i}\frac{D_{i}}{t_{i}}}{\sum_{i=1}^{M}w_{i}}\;,$$

with

(8) $$t_{1}>t_{i\;}>t_{M}\;,$$

(9) $$D_{max}>D_{1}>D_{i\;}>D_{M}\;,$$

where *M* is the number of acquisitions, *D_i_* is the pixel value of the *i*th image, and *D*_max_ is the pixel saturation threshold. The reduction factor of integration time between two successive images of the LDR series can be expressed as:

(10) $$f_{t}=\frac{t_{i}}{t_{i+1}}\;.$$

By uncertainty propagation as described by the Guide to the Expression of Uncertainty in Measurement (GUM [20]), the uncertainty of the luminance can be derived as:

(11) $$u\left(L_{w}\right)=\frac{\sqrt{\sum_{i=1}^{M}{w_{i}}^{2}\frac{u^{2}\left(D_{i}\right)}{{t_{i}}^{2}}}}{\sum_{i=1}^{M}w_{i}}.$$

where only the uncertainty of the variable *D* is considered and only its random component, or noise. There is no uncertainty associated with the weighting factors, since they are part of the definition of the HDR merging method. In addition, no uncertainty on integration time is considered for this analysis.

To evaluate the performance of the method, we evaluate how much the luminance uncertainty decreases with respect to the case where the optimal integration time for each pixel is used for its calculation—corresponding to applying the best exposure algorithm described in Section 2 (*i* = 1 and *M* = 1, where the integration time has the maximum value before saturation). This can be expressed as:

(12) $$\frac{u(L_{w})}{u(L_{1})}=\frac{\sqrt{\sum_{i=1}^{M}{w_{i}}^{2}\frac{u^{2}(D_{i})/u^{2}(D_{1})}{{t_{i}}^{2}/{t_{1}}^{2}}}}{\sum_{i=1}^{M}w_{i}}.$$

As the only error considered is shot noise, we have:

(13) $$\frac{u^{2}(D_{i})}{u^{2}(D_{1})}=\frac{t_{i}}{t_{1}}={f_{t}}^{1-i}\;.$$

Hence, we obtain the following expression for the general uncertainty reduction:

(14) $$\frac{u(L_{w})}{u(L_{1})}=\frac{\sqrt{\sum_{i=1}^{M}{w_{i}}^{2}{f_{t}}^{i-1}}}{\sum_{i=1}^{M}w_{i}}\;.$$

This uncertainty reduction factor can be examined for different weighting factors. Since acquisitions with larger SNR should count more in the weighted average, we propose to evaluate the weight as exponential functions of the SNR: SNR^2^, SNR^1^, and SNR^0^ (or uniform weighting). It must be noticed that the SNR is proportional to the square root of the number of photoelectrons, and consequently to the square root of the integration time. Therefore, we obtain *w_i_* = *t_i_*, in the case of SNR^2^ and *w_i_* = $\sqrt{t_{i}}$, in the case of SNR^1^. Using these weight factors in Equation (14), we obtain the following expressions:

- Case of SNR^2^:

(15) $$\frac{u(L_{w})}{u(L_{1})}=\frac{\sqrt{\sum_{i=1}^{M}{f_{t}}^{1-i}}}{\sum_{i=1}^{M}{f_{t}}^{1-i}}=\frac{1}{\sqrt{\sum_{i=1}^{M}{f_{t}}^{1-i}}}\;.$$

- Case of SNR^1^:

(16) $$\frac{u(L_{w})}{u(L_{1})}=\frac{1}{\sum_{i=1}^{M}\sqrt{\frac{{f_{t}}^{1-i}}{M}}}\;.$$

- Case of SNR^0^:

(17) $$\frac{u(L_{w})}{u(L_{1})}=\frac{\sqrt{\sum_{i=1}^{M}{f_{t}}^{i-1}}}{M}\;.$$

**Figure 2:**
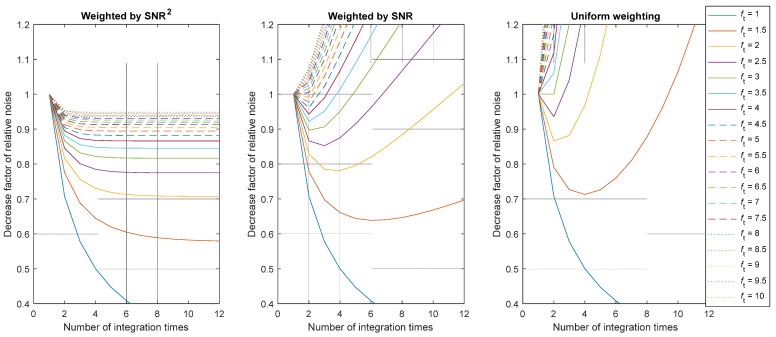
Value of the uncertainty reduction factor according to the number of combined images. Plots given for various integration time reduction factors *f*_t_ and for the three considered weights (SNR^2^, SNR or 1). Note that in practice, the number of combined images is limited due to saturation.

**Figure 3:**
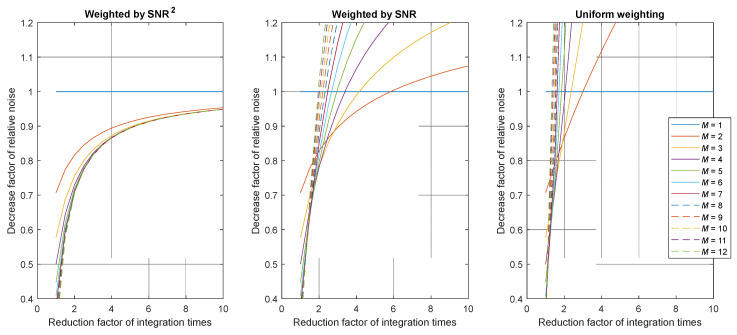
Value of the uncertainty reduction factor as a function of the integration time reduction factor. Plots given for various numbers of images *M* and for the three considered weights (SNR^2^, SNR, or 1).

In all cases, the reduction depends on the variables *M* and *f*_t_. The representation of the functions given in Equations (15)–(17) is shown in Figure 2 and Figure 3. An uncertainty reduction factor below 1 means that the SNR of the HDR pixel is increased using the tested algorithm compared to using the best exposure algorithm.

Figure 2 and Figure 3 show that only a weighted HDR algorithm with an SNR^2^ as a weighting function would improve the SNR of the resulting measurement, as the value of the uncertainty reduction factor is always below 1. However, this improvement is below 15% in the reduction of the relative noise for $f_{t}>4$. Using the weighting function SNR^2^, the pixel SNR can almost be doubled in the best case. In the specific case where *f*_t_ = 1, which is the singular case where there is no variation in the integration time, the pixel value is averaged over several identical captures, significantly increasing the SNR but without increasing the measurable dynamic range, which defies the purpose of HDR imaging.

The HDR imaging method based on weighted average in the logarithmic space (see Equation (4)) can be studied using a similar method to compare if it reduces uncertainty with respect to the linear approach, which yields similar results. From this analysis, we can conclude that, if the only source of error is shot noise:

- A weighting factor proportional to SNR^2^ is more efficient for the reduction of the noise. This weighting factor corresponds with using *t_i_*, *N,* or *N*^2^/*t_i_*. We think that the more convenient weight to be used is *t_i_*, because using *N* within this factor would add unnecessary noise to the method.
- The use of *t_i_* as a weighting factor would reduce the uncertainty by 30% and 15%, respectively, in the practical cases of *f*_t_ = 2 and *f*_t_ = 4, with respect to the case where the optimal integration time is used for each pixel.
- The logarithmic approach does not improve the reduction in uncertainty in this shot-noise-limited case.

This first evaluation guided us in choosing the tested weight factor. However, the simplistic hypothesis applied in this section does not account for all the parameters that may impact the performance of an HDR imaging method. The numerical evaluation based on the modeling of a camera “digital twin”, detailed in the next section, allows us to evaluate the performance of an HDR imaging method based on a more realistic camera.

## 4. Evaluation Method

**Figure 4:**
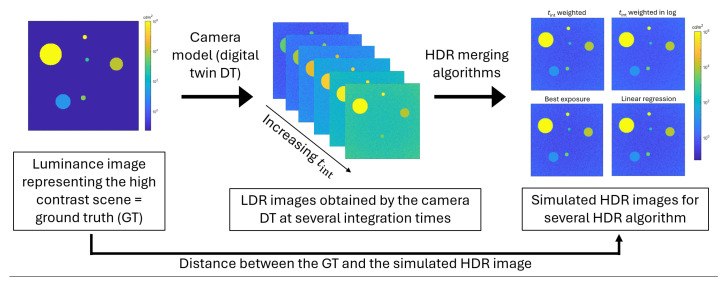
Overview of the HDR imaging method evaluation process.

The evaluation framework developed to study HDR imaging methods, illustrated in Figure 4, comprises four main elements: a high contrast ground-truth virtual luminance scene (GT), a digital twin (DT) of the camera response, HDR merging algorithms, and evaluation metrics used to compare the performance of the different HDR imaging methods. The DT generates synthetic LDR images from the GT according to the chosen camera and acquisition parameters. These LDR images are then combined using the tested HDR merging algorithms presented in Section 2.2 [21] to produce HDR images. The performance of the HDR imaging method is finally quantified by comparing the GT with the resulting HDR images using metrics tailored to our needs.

### 4.1. Digital Twin (DT) of the Camera Response

The response of arbitrary and conventional luminance cameras was simulated by considering the known error sources, that is, the effects that cause the camera response to deviate from the true luminance to be measured. This simulation, referred to hereafter as the “DT response”, enables the generation of different camera behaviors by adjusting the parameters associated with these error sources. As such, the DT response is a highly suitable tool for systematically assessing how various error sources affect the performance of each HDR imaging method under evaluation.

**Figure 5:**
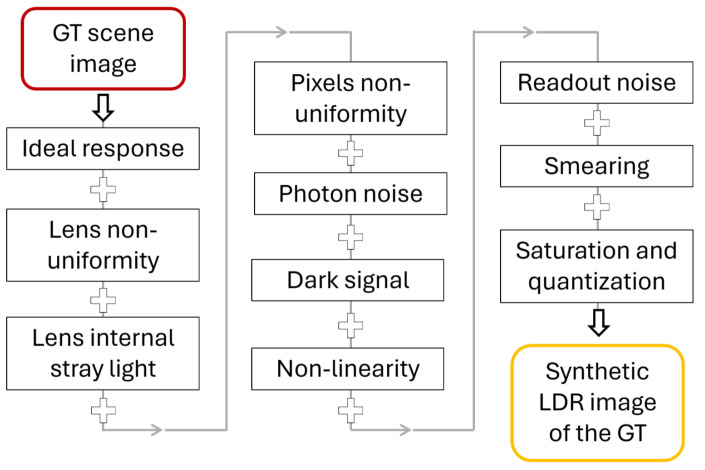
The principle of the camera response proposed in [22], from which our model is based. The effects applied on the GT scene to model a synthetic LDR image must be applied in a specific order, indicated by the arrows.

The DT response is based on the principle proposed in [22] and illustrated in Figure 5. The included error sources are photon noise, readout noise, dark signal, photo-response non-uniformity (PRNU) of the array sensor, lens-shading non-uniformity, non-linearity, internal stray light, smearing, saturation, and adjustment of the luminous responsivity. These error sources are implemented using a parametrized function and random values in the case of noise-related effects, and they are applied sequentially in a specific order. In the evaluation presented here, some effects that introduce systematic errors independently of the measurement method (HDR or LDR imaging) were removed to simplify the problem and focus on the most relevant parameters. This also reduces the computational cost of the evaluation, particularly by omitting effects modeled as image convolutions (e.g., internal stray light, which is represented by a point spread function applied to the image).

The camera model used in the evaluation method includes the following effects, applied sequentially:

1. Ideal response–The ideal response applied to each pixel of the GT [cd·m^−2^] considers a linear camera of luminous responsivity *s*_V_ [count·ms^−1^·cd^−1^·m^2^]. The response is proportional to the target integration time *t*_int_ [ms], which may be impacted by a systematic error *εt*_int_ [ms]:

(18) $$R_{1}=GT\cdot \tilde{t_{int}}\cdot s_{V}\;\;with\;\tilde{t_{int}}=t_{int}+\varepsilon t_{int}\;.$$

For HDR capture, the target integration time of the *i*th image of the simulated LDR series is defined within the range of possible integration times [*t*_min_: *t*_max_] based on the integration time reduction factor *f*_t_ as:

(19) $$t_{int}\left(i\right)=\frac{t_{max}}{{f_{t}}^{i-1}}\geq t_{min}.$$

The next image is captured as long as there are still saturated pixels on the image, under the condition that the integration time remains above the shortest possible integration time *t*_min_.

2. Photon noise–The randomness associated with photons arriving at the detector results in a Poisson distributed noise, whose standard deviation is equal to the square root of the average number of electrons, and therefore depends on the overall “gain” of the sensor *K* [count·electron^−1^] (inversely proportional to the ISO) [17]. With *randn,* a random value obtained from a standard normal distribution, we have for each pixel:

(20) $$R_{2}=R_{1}+\frac{\sqrt{K\cdot R_{1}}}{K}\;randn\;.$$

3. Dark signal–An electrical signal is produced by a camera sensor even in complete darkness. It is caused by the thermal generation of electrons within the semiconductor material during the integration time, photon noise of the dark, and some residual offset. With *r*th the rate of thermally produced counts [count·ms^−1^] and *D*_offset_ [count], a constant offset, the dark signal is applied as:

(21) $$R_{3}=R_{2}+D_{offset}+r_{th}\cdot \tilde{t_{int}}+r_{th}\frac{\sqrt{K\cdot r_{th}\cdot \tilde{t_{int}}}}{K}\;randn\;.$$

**Figure 6:**
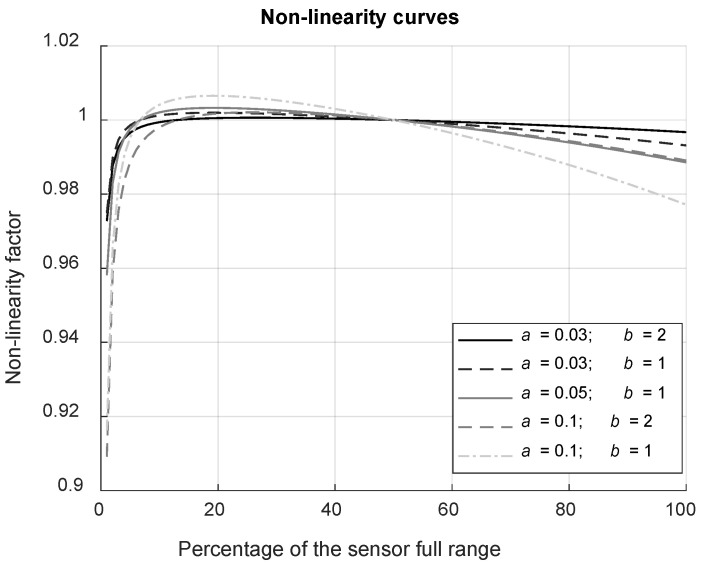
Curves representing the multiplying factor applied to model the non-linearity behavior of the sensor as a function of the pixel value expressed in percentage of the sensor’s dynamic range. The different curves, ranging from the smallest non-linearity to the largest non-linearity, correspond to the five models applied during testing (see Section 4.4).

4. Non-linearity–Although the camera is considered linear, pixel values do not increase exactly proportionally to the incident irradiance, especially for low count values and near saturation. The function *f*_NL_ proposed to model non-linearity, illustrated in Figure 6, has been chosen empirically for its potential to mimic plausible effects while depending on only two parameters, *a* and *b*:

(22) $$f_{NL}\left(x\right)=1-a\left(\frac{dyn}{100x}+\frac{e^{\frac{x}{dyn}-1.5}}{b}\right)+a\left(\frac{1}{50}+\frac{e^{-1}}{b}\right)\;with\;dyn=2^{Nbits}-1\;.$$

where *x* is a count value within the dynamic range of the sensor described by the bit depth *Nbits*. The non-linearity is applied as a multiplicative factor for each pixel:

(23) $$R_{4}=R_{3}\cdot f_{NL}(R_{3})\;.$$

5. Readout noise–The variance in counts from repeated readings of a pixel is considered using a random noise of standard deviation *σ*_read_ [count]:

(24) $$R_{5}=R_{4}+\sigma_{read}\cdot randn\;.$$

6. Saturation and quantization–Pixel values are limited to integers within a range limited by the bit depth of the sensor *Nbits*, above which saturation occurs. With [.] the floor rounding operation, we have:

(25) $$R_{6}=\min\left(\left[R_{5}\right],2^{Nbits}-1\right)\;.$$

Using the DT response described by Equations (18) to (25), synthetic LDR images at different integration times can be obtained and merged into an HDR image applying an HDR imaging method. The comparison of these output images with the input image (GT) allows the evaluation of the performance of the studied HDR approaches.

### 4.2. Definition of the Virtual High Contrast Luminance Scenes (Ground Truth)

**Figure 7:**
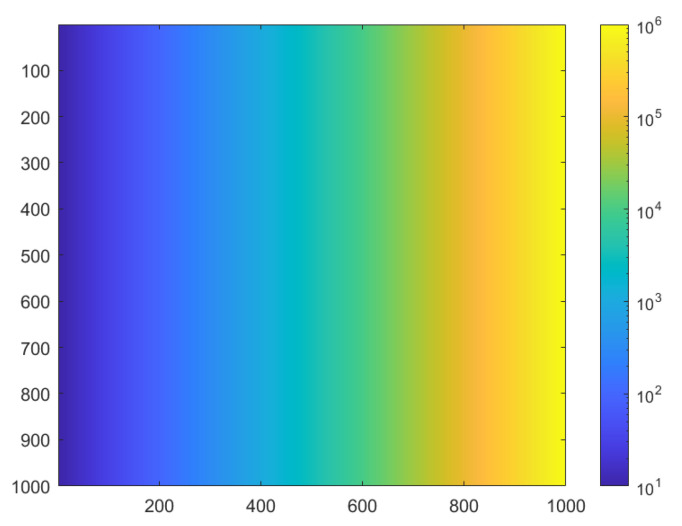
Luminance image representing an example of a gradient luminance scene used to evaluate the HDR imaging parameters impact using a pixel-by-pixel approach. Here, the luminance ranges from 10 cd/m^2^ to 10^6^ cd/m^2^, but these lower and upper limits can be modified according to the testing needs.

The impact of the HDR imaging method parameters can be classified into two categories: some parameters influence the value of a pixel independently of the surrounding pixels, while others impact the image spatially. In this study, we limited our evaluation to the former parameters. For this purpose, it is essential to test a wide range of luminance values; however, the inclusion of specific spatial frequency content is unnecessary. The proposed virtual ground truth (GT) scene, shown in Figure 7, consists of a horizontal luminance gradient, where each column corresponds to a luminance value within a customized range, distributed according to a logarithmic progression. The number of columns defines the number of luminance levels tested, while the number of rows corresponds to the sampling size, enabling a statistical analysis of noise.

Temporal instability in scene luminance can affect HDR luminance measurements. For example, fluctuations from a very bright source may significantly impact its measured value because high luminance levels require short integration times. As a result, these fluctuations are less likely to be averaged out, unlike fluctuations from a low luminance source, which are measured using longer integration times and therefore benefit from temporal averaging. The proposed HDR merging algorithms may be sensitive to luminance temporal fluctuations, as they combine the images measured at different instances differently. To study the impact of source temporal fluctuations, the effect has been modeled as a multiplying factor on the GT image as part of the camera digital twin (DT):

(26) $$GT=GT\cdot \left(1+\;\sigma_{source}\frac{\sum randn\left(T\right)}{T}\right)\;\;with\;T=\left[\frac{t_{int}}{\Delta t_{source}}\right]\;.$$

where *σ*_source_ is the standard deviation of the source fluctuations observed with a temporal period of Δ*t*_source_, *t*_int_ is the integration time during which the source is acquired by the camera, *randn*(*T*) designates *T* random samples distributed according to a normal distribution, and [.] designates floor rounding.

### 4.3. Evaluation Metrics

The performance of the tested HDR methods is evaluated according to criteria relevant to luminance measurement in lighting applications. Although accurate rendering of spatial information is essential, the primary focus is on the accuracy of the measured luminance values and the level of noise observed in regions of interest, typically corresponding to groups of image pixels. The good reproduction of image gradients (without discontinuities related to HDR reconstruction) is also considered important, but has not been specifically tested here, as it is redundant with evaluating the accuracy of the luminance values. Finally, the ability of the method to reproduce the widest possible dynamic range is a key criterion, including the capability to accurately measure very dark regions despite the presence of high luminance areas within the camera’s field of view. The metrics typically proposed to quantify distance between images [23] are not sufficiently adapted to our objectives. Parameters that impact only the pixel values of an image can be evaluated using metrics that are not specific to image processing. Therefore, we derived metrics based on classical signal processing to evaluate the distance between the GT and the simulated HDR images, which we call bias and noise.

The HDR image accuracy in terms of average luminance level is characterized by the bias, which we define as the relative difference between the GT value and the average value obtained on the HDR image. The HDR image quality in terms of noise, referring to the random fluctuations around the average value, is characterized by the relative standard deviation of the values corresponding to the same luminance level.

On the gradient scene, the bias *b_i_* for the column of index *i* is computed as the relative difference between the GT value of the column *GT_i_* and the average luminance value of the simulated HDR image (*L*_HDR_) for the same column. The noise *ε_i_* is computed as the standard deviation over the pixel values of the column, divided by the average value over the column. With ⟨⋅⟩*_i_* referring to the averaging operation over all pixels in column *i*, we have:

(27) $$b_{i}=\;\frac{{\langle L_{HDR}\rangle }_{i}-GT_{i}}{GT_{i}},$$

(28) $$\varepsilon_{i}=\frac{{\sqrt{\langle (L_{HDR}-{\langle L_{HDR}\rangle }_{i}{)}^{2}\rangle }}_{i}}{{\langle L_{HDR}\rangle }_{i}}\;.$$

### 4.4. Tested Parameters and Strategy

The parameters varied during the tests can be described as camera properties (parameters, noises and errors), acquisition parameters, HDR merging algorithm parameters, and scene properties. They are listed in Table 1, Table 2, Table 3 and Table 4.

A reference camera model, representing a good quality camera with relatively low noise levels, has been defined using the parameter values highlighted in bold in the tables. For each parameter, additional values corresponding to different noise levels or different performance levels have been specified.

The proposed parameters are inspired by three main hardware categories: scientific cameras (we use an ORCA Flash 4.0 v3 with a −10 °C cooled CMOS sensor from Hamamatsu), industrial cameras (we tested the acA2040-90mNIR model from Basler), and consumer cameras (such as Nikon and Canon DSLRs).

According to the literature, integration time errors are well below one millisecond for most models and can be as small as a few microseconds, as the internal clock is generally well controlled. We therefore considered small values in our study, although mechanical shutters may lead to larger deviations.

The proposed rates of thermally generated counts (or electrons, for a gain equal to 1) are in the upper range compared with data published by manufacturers. For cooled sensor sensors, this rate is very low (6 × 10^−5^ electrons·ms^−1^ for our ORCA camera), and below 1 × 10^−3^ electrons·ms^−1^ for DSLRs at room temperature. However, we surprisingly measured 0.25 electrons·ms^−1^ for our Basler camera (not equipped with a heat-sink) in normal ambient conditions, with even worse performance for integration times longer than 500 ms.

Finally, the mathematical model proposed to describe non-linearity was empirically determined to reproduce the general trends observed in experimental measurements. The non-linearity values measured experimentally are about 2% (following the EMVA method [17]) for the Basler camera (corresponding to *a* = 0.1 and *b* = 1), and about 0.5% for the ORCA camera (approximately *a* = 0.03 and *b* = 1). This model is also suitable to represent residual non-linearity after correction (e.g., 0.5% and 0.2% for the Basler and ORCA cameras mentioned above, respectively).

The impact of a given parameter on the HDR luminance measurement performance is evaluated by considering a camera model in which all the parameters correspond to the reference except for the parameter being tested, which takes the range of values indicated in the tables. This approach allows us to analyze the sensitivity of the luminance measurement to each parameter individually, and to determine whether any of the four tested HDR algorithms performs better under certain conditions.

For some specific parameters, the reference model is not adequate for testing. In that case, a tailored reference is used. For example, the choice of threshold values for the HDR merging algorithm may represent a loose constraint, a moderate constraint, and a strict constraint (see Table 3). To better observe the impact of the threshold, some non-linearity must be introduced in the camera model.

For all the tests, the luminance range of the gradient GT scene spans 6 decades, from 0.1 cd·m^−2^ to 100,000 cd·m^−2^.

## 5. Simulation Results

The simulation results obtained through applying the testing method are detailed in this section. Each parameter, describing the camera properties (error on the integration time, photon noise, dark signal readout noise, non-linearity, and sensor bit-depth), the HDR method parameters (integration time reduction factor, maximum and minimum integration times, HDR algorithm thresholds), and the scene stability, has been analyzed separately.

### 5.1. Effects of Camera Properties

#### 5.1.1. Error on the Integration Time

**Figure 8:**
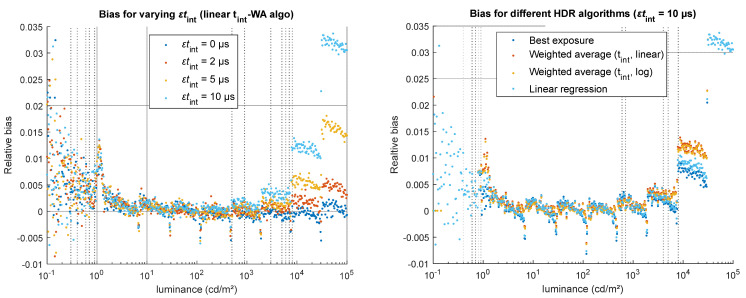
Impact of a constant error on the integration time (*εt*_int_) on the accuracy of the simulated HDR luminance values. (**Left**) for one HDR algorithm (linear t_int_-WA), relative bias obtained for four values of *εt*_int_ (0, 2, 5, and 10 µs). (**Right**) for a high error (*εt*_int_ = 10 µs), relative bias obtained using the four HDR merging algorithms.

A constant error in the integration time impacts the bias, as illustrated in Figure 8, but does not impact the noise. We observe a strong increase in the bias for high luminance levels. The increase is linear to the parameter value: a double error on the integration time yields to a double bias for the same luminance level. Regarding the impact of the choice of the HDR merging algorithm (Figure 8, right), the best exposure (BE) and linear regression (LR) algorithms are less sensitive to this error than the integration time weighted average (t_int_-WA) algorithms (in the linear and log domains).

#### 5.1.2. Photon Noise

**Figure 9:**
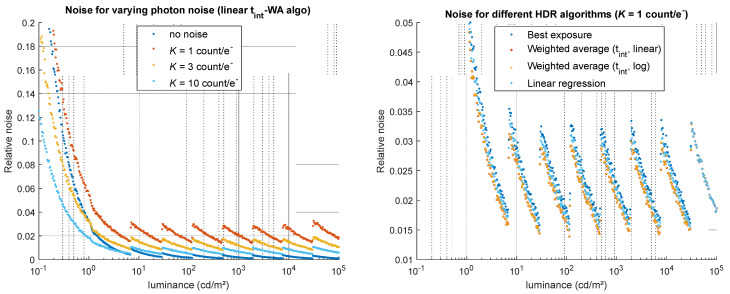
Impact of photon noise (related to the camera’s overall gain *K*) on the noise of the simulated HDR luminance values. (**Left**) for one HDR algorithm (linear t_int_-WA), relative noise obtained for four values of *K* (no noise, 1, 3, and 10 count/e^−^). (**Right**) for the highest noise (*K* = 1 count/e^−^), relative noise obtained using the four HDR merging algorithms.

Photon noise obviously impacts the relative noise computed on the simulated HDR image, inversely proportional to the camera’s overall gain *K* (the higher *K*, the lower the noise). A very small impact on bias is observed as well, probably due to the fact that the parameter *K* also impacts photon noise on the dark signal. As shown on Figure 9, the noise magnitude is significantly high for low luminance values but remains in a similar range for the rest of the luminance range. t_int_-WA algorithms are less sensitive to this noise than BE and LR algorithms by roughly 20% (Figure 9, right—note that the noise for both WA algorithms is identical here), which is coherent with the theory presented in Section 3.

#### 5.1.3. Dark Signal

**Figure 10:**
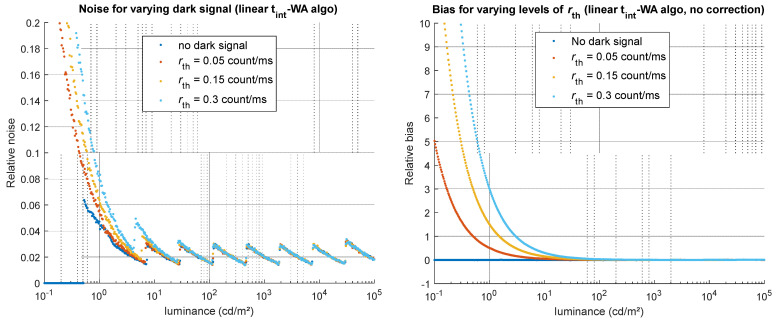
Impact of *r*_th_ on the simulated HDR luminance values (obtained using the linear t_int_-WA algorithm). (**Left**) relative noise obtained for four values of *r*_th_, whether a dark correction is applied or not. (**Right**) relative bias obtained for four values of *r*_th_ when no dark correction is applied.

To evaluate the impact of the dark signal, tests were conducted using the HDR code with the dark correction step either enabled or disabled. When no dark correction is applied, the dark signal, composed of an offset, thermally generated counts, and the associated shot noise, strongly impacts the measurement of low luminance levels with noise and bias. The noise is principally related to the thermal rate, which yields relative noise ranging from roughly 5% to 20% near the smallest measurable values (below 1 cd/m^2^) on our test, illustrated in Figure 10. The bias, also mainly due to thermally generated counts, can get extremely high at low luminance values if no correction is applied: on the tests performed for various thermal rate values, we observe for a luminance of 1 cd/m^2^ a positive bias (luminance is overestimated) ranging from 60% to 300% when no dark correction is applied (Figure 10, right).

When a median dark correction is applied (the median of the dark image is subtracted, with the condition that the dark images used for the correction are captured exactly in the same conditions as the LDR images), the dark offset seems to have little impact on the bias and on the noise. As for the dark signal originating from thermally generated electrons, little effect is observed on the bias when a dark correction is applied (except for very low luminance values, corresponding to very long integration times), but the effect on the noise is strong, identical to when no dark correction is applied (see Figure 10, left).

For these sources of noise and error, the choice of algorithms has no visible influence.

#### 5.1.4. Readout Noise

**Figure 11:**
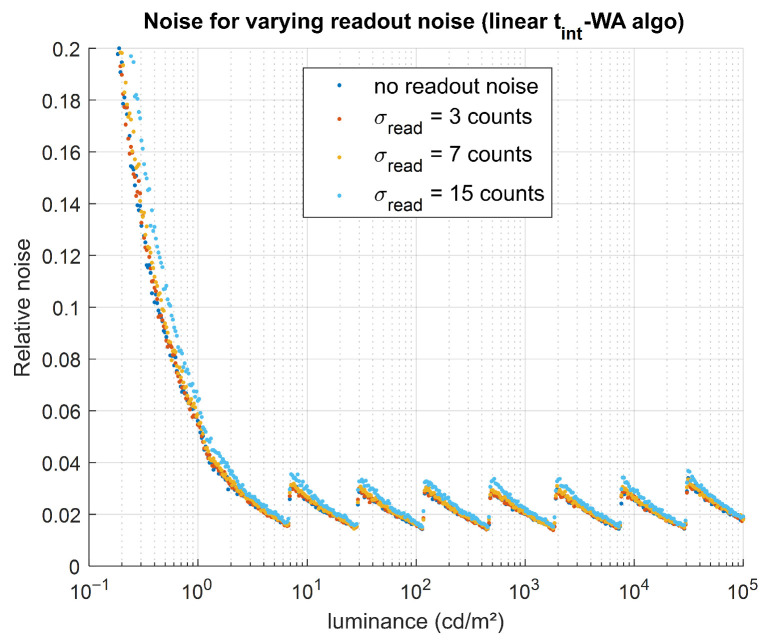
Impact of readout noise (related to *σ*_read_) on the simulated HDR luminance values. For one HDR algorithm (linear t_int_-WA), relative noise was obtained for four values of *σ*_read_ (0, 3, 7, and 15 counts).

Readout noise has little impact on bias (no noticeable effect when a dark correction is applied, and a negligible effect compared with the impact of dark signal when no correction is applied) and only a very small impact on noise. This is illustrated in Figure 11, where the increase in noise with increasing readout noise remains small relative to the noise level caused by other effects. No HDR algorithm appears to be notably more robust than the others.

#### 5.1.5. Sensor’s Non-Linearity

**Figure 12:**
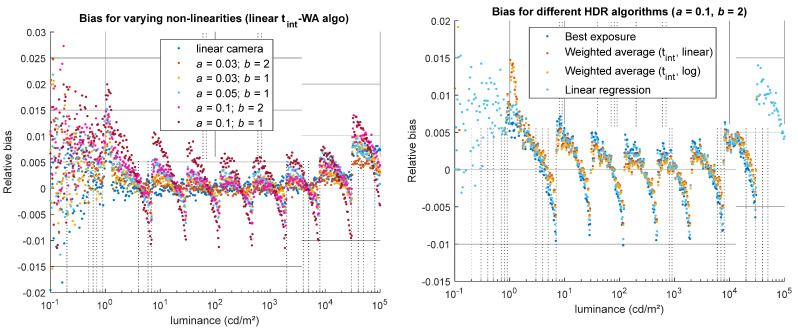
Impact of non-linearity (parameters *a* and *b*) on the bias of the simulated HDR luminance values. (**Left**) for one HDR algorithm (linear t_int_-WA), relative bias obtained for a linear camera and 5 models on non-linearity. (**Right**) for a high non-linearity (*a* = 0.1, *b* = 2), relative bias obtained using the four HDR merging algorithms.

Non-linearity does not affect noise; however, it can strongly impact the accuracy of the HDR luminance (bias). Its influence depends primarily on the specific non-linear behavior of the sensor, and to a lesser extent on the type of HDR-merging algorithm. For the best exposure algorithm, the non-linearity error present in the LDR image is directly propagated into the HDR result. For algorithms that combine multiple LDR images, the bias due to non-linearity is often slightly reduced, provided that the HDR algorithm thresholds are appropriately set. Our tests, illustrated in Figure 12, show that the t_int_-WA algorithms are slightly less sensitive to this type of error than the BE and LR algorithms, as the absolute value of the relative bias his slightly lower for these WA methods. However, it should be emphasized that this result is exclusive to the particular type of non-linear behavior applied in this evaluation.

#### 5.1.6. Sensor’s Bit Depth

**Figure 13:**
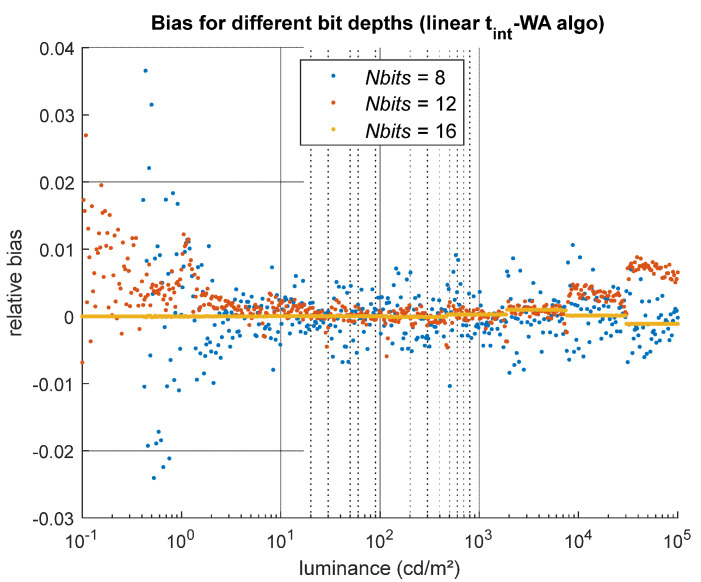
Impact of quantization only on the bias of the simulated HDR luminance values. For this source of error, which depends on the sensor bit depth *Nbits*, it is the standard deviation of the relative bias between the different luminance levels that informs us of the impact of this parameter.

Tests were performed to assess the influence of the sensor bit depth (8, 12, or 16 bits) on the HDR results. For these tests, the luminous sensitivity of the camera was adjusted so that the same luminance range could be captured regardless of the sensor bit depth, and all the other sources of noise were disabled in the camera model to observe only the impact of quantization on the HDR results. The effect of quantization is most visible in the standard deviation of the relative bias across different luminance levels (i.e., across the columns of the GT and HDR images). As shown in Figure 13, the relative bias barely fluctuates between luminance values for a 16-bit camera (yellow dots), while the fluctuations increase for 12-bit and 8-bit cameras (orange and blue dots, respectively). Between 20% and 80% of the GT dynamic range, the bias fluctuates with a standard deviation of 0.13% for the 12-bit camera and 0.33% for the 8-bit camera, values that remain small compared with other sources of error. However, at low luminance levels, the quantization error can reach up to 2% and 4% for 12-bit and 8-bit cameras, respectively. The sensor bit depth also limits the measurable dynamic range: in our test configuration, very low luminance values cannot be measured with an 8-bit camera, likely because insufficient light reaches the sensor to produce at least one count.

### 5.2. Effects of HDR Method Parameters

#### 5.2.1. Integration Time Reduction Factor

**Figure 14:**
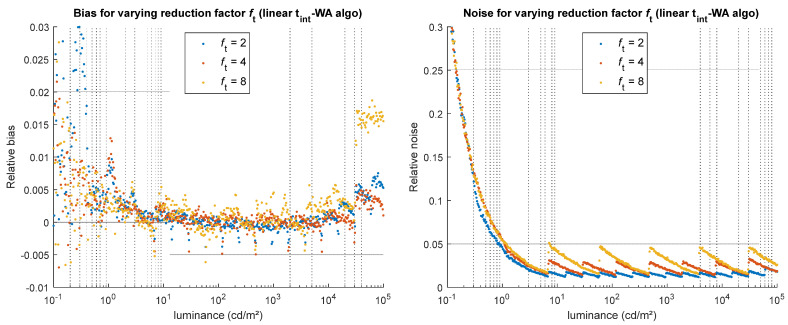
Impact of the integration time reduction factor *f_t_* on the simulated HDR luminance values, for the linear t_int_-WA algorithm. (**Left**) relative bias obtained for various *f_t_* values. (**Right**) relative noise obtained for various *f_t_* values.

Three reduction factors were tested using our method: 2, 4, and 8. As illustrated on Figure 14, the higher the reduction factor, the higher the noise and bias, which result from all the camera errors that are less reduced by the HDR merging when there are fewer LDR images combined together. This effect, which is significant in noise when the reduction factor is high (several percent for *f_t_* = 8 in the example shown in Figure 14 right), is similar regardless of the HDR merging algorithm.

The choice of the time reduction factor between consecutive exposures reflects a compromise between dynamic range expansion, noise performance, and acquisition time constraints. When total acquisition time and scene stability are not limiting factors, a small reduction factor can be used to optimize noise performance. The required dynamic range can then be achieved by capturing as many images as necessary. The maximum attainable dynamic range is, however, ultimately limited by hardware properties (mainly, thermal noise at long integration times and, although not addressed in this study, stray light). In situations where acquisition time, motion, or storage constraints apply, a larger reduction factor reduces the number of required exposures and shortens the total acquisition time, while still allowing the dynamic range to cover that of the scene of interest. However, the cost of using larger reduction factors is having a larger noise in the intermediate signal range of the HDR image. In such cases, the impact of noise can be partially mitigated through spatial averaging when this is compatible with the application.

#### 5.2.2. Minimum and Maximum Integration Times

The minimum and maximum integration times (*t*_min_, *t*_max_) determine the measurable dynamic range. A short minimum integration time makes it possible to measure high luminance values, provided that the integration time is accurately known, since, as shown in 5.1, inaccuracies in the integration time can lead to high errors for high luminance levels. Similarly, a long maximum integration time enables the measurement of low luminance values, assuming the camera’s thermal noise is not too high, in which case noise at low luminances can become significant.

#### 5.2.3. Algorithm Thresholds

**Figure 15:**
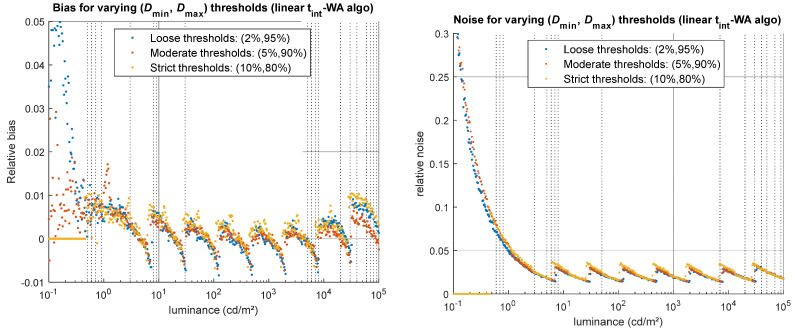
Impact of the choice of HDR merging algorithm’s thresholds on the simulated HDR luminance values, for the linear t_int_-WA algorithm. (**Left**) relative bias obtained for more or less strict thresholds. (**Right**) relative noise obtained for more or less strict thresholds.

To assess the impact of the HDR merging algorithm’s threshold parameters on the resulting HDR image, three threshold levels—considered as loose, moderate, and strict—were tested for a camera presenting a relatively high non-linearity (*a* = 0.1, *b* = 2). The results, illustrated in Figure 15, show that overly strict thresholds surprisingly lead to increased noise and bias, as well as a reduced dynamic range (no HDR values are computed for some luminance levels in the first decade of the GT range, as shown by the 0 values indicated in Figure 15–yellow dots). Overly loose thresholds produce more bias at low and high luminance values, but lower noise overall, since the high pixel values associated with a high SNR are used in the HDR merge. The moderate thresholds perform the best in this case, producing less noise than the strict thresholds, less bias than the loose thresholds, and maintaining a relatively wide dynamic range. The type of HDR merging algorithm employed has little effect here.

The choice of appropriate thresholds can be guided by the camera properties. The upper threshold can be determined based on an experimental measurement of the camera’s non-linearity. It can be set as high as possible, up to the point where the non-linearity reaches a maximum acceptable percentage, corresponding to the larger bias the user can accept. Since it is often difficult to experimentally characterize camera behavior very close to clipping or saturation, the highest upper threshold allowed for a camera with well-corrected non-linearity rarely exceeds 95% of the sensor’s dynamic range. The choice of a lower threshold involves a trade-off between noise, bias, and the reconstruction of low luminance values (i.e., dynamic range). Knowledge of dark signal and thermal noise, although dependent on integration time, can help estimate the minimum pixel value below which noise dominates, thereby providing an order of magnitude for selecting an appropriate lower threshold.

### 5.3. Effects of Scene Stability

**Figure 16:**
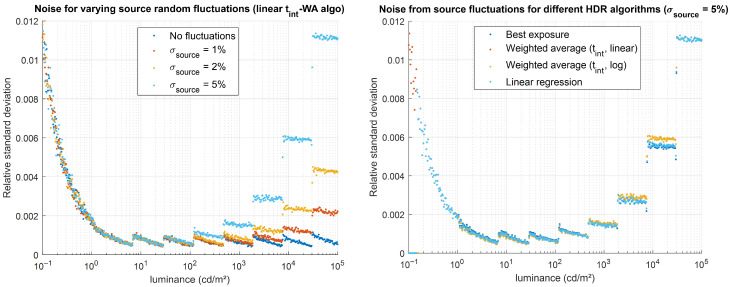
Impact of source temporal fluctuations (described by a normal distribution of standard deviation *σ*_source_) on the simulated HDR luminance values. (**Left**) relative noise obtained for various values of *σ*_source_, for the linear t_int_-WA algorithm. (**Right**) relative noise obtained for different HDR algorithms for *σ*_source_ = 5%.

The impact of a random luminance-source instability (white noise) was evaluated by applying the evaluation method to the gradient GT with a Monte Carlo approach, with 100 independent noise realizations. This allowed us to statistically evaluate how random temporal fluctuations of the luminance source propagate into the HDR measurement by calculating the relative standard deviation across these 100 simulations. Four values for the standard deviation of the source temporal fluctuations were tested, ranging from no noise to 5%, with a temporal periodicity of Δ*t*_source_ = 10 µs. The evaluation results are shown in Figure 16. As predicted by theory, the temporal fluctuations of the source propagate into the HDR luminance measurement, producing noise that depends both on the standard deviation of the source fluctuations and on the integration time required to capture the LDR image used to determine the luminance at each level: for a normal distribution, we roughly obtain a noise of standard deviation $\sigma =\;\sigma_{source}/\sqrt{t_{int}/∆t_{source}}$ for an LDR image acquired with an integration time *t*_int_.

The tests also show that the best exposure algorithm and linear regression algorithms are slightly more robust to this effect, although the noise difference between the different HDR algorithms remains small (see Figure 16 right). When this type of error affects HDR imaging, alternative methods may be considered to mitigate noise at high luminance values. For example, capturing multiple images at short integration times and averaging them would improve the resulting HDR luminance measurement without excessively increasing the overall acquisition time.

## 6. Discussions and Conclusions

Following a theoretical study that allowed us to identify the type of HDR algorithm most likely to deliver reliable results (Section 3), we conducted a simulation-based evaluation using a camera model. This enabled us to analyze the impact of several parameters characterizing the camera, the image-acquisition method, the HDR reconstruction method, and the properties of the scene. By evaluating the influence of each parameter, we were able to observe their effects in terms of random error (noise), systematic error (bias), and the dynamic range of the acquired image.

The parameters associated with the camera and the scene introduce varying levels of noise or bias. Inaccuracies in the integration time (*εt*_int_) and sensor non-linearity introduce only bias: limited to high luminance values for *εt*_int_, and present across the entire luminance range for non-linearity. Conversely, photon noise, dark signal (when a dark correction is applied), readout noise, and quantization all affect the noise in the HDR image. This noise is very high at low luminance levels and then typically stabilizes within a given range for higher luminance values, with photon noise dominating the overall behavior.

The HDR-method parameters tested show results that are strongly correlated with the camera characteristics. For example, the choice of a small integration time reduction factor can minimize the impact of camera noise only when the scene’s temporal stability is sufficient, as the overall measurement time increases. Short minimum and long maximum integration times also help improve the dynamic range of the HDR image, provided that noise related to integration-time accuracy, thermal photon generation, or scene instability does not degrade the captured information. Finally, the study of HDR-algorithm thresholds showed that overly strict thresholds are counterproductive: the best strategy is to select thresholds that are as loose as possible (i.e., as close as the minimum and maximum possible values) while keeping non-linearity within an acceptable range. To make an informed choice, it is therefore necessary to know the sensor’s non-linearity or, at least, to have an estimate of the residual non-linearity according to the pixel value when a non-linearity correction is applied.

The tested algorithms exhibit only slightly different performance depending on the type of noise. The best exposure (BE) and linear regression (LR) algorithms perform marginally better than the time weighted average (t_int_-WA) algorithms in the presence of integration-time errors and readout noise. They also show greater robustness when the scene luminance fluctuates. For other error sources (photon noise, dark signal, and non-linearity), the t_int_-WA algorithms provide slightly better results, especially in terms of signal-to-noise ratio (SNR) of the calculated HDR luminance values. However, these differences remain generally small and relatively insignificant compared with the magnitude of the overall HDR luminance measurement error. The order of magnitude of the error remains essentially unchanged regardless of the algorithm used, and the optimal algorithm strongly depends on the type of camera used, with a better SNR generally obtained using the t_int_-WA algorithm, but a possibly lower systematic error using the BE algorithm. A noteworthy exception is the case of a systematic integration-time error, for which the BE and LR algorithms can help reduce the resulting systematic bias. From these observations, criteria other than result accuracy can be considered to choose the most suitable HDR merging algorithm, such as implementation simplicity or easy calculation of uncertainty propagation. For applications where simplicity and accuracy are essential, and where SNR is not the most important criterion, the best-exposure algorithm can be recommended.

Our tests also show that a good dark correction is required for accurate measurements for low luminance levels, and that thermal rate can significantly limit the smallest measurable luminance value by impacting the noise at low luminance levels. As this rate is linked to the sensor’s temperature, using a heat sink can be an easy way to improve HDR measurement results when the sensor is prone to heating. The tests also show that errors can be easily reduced by increasing the number of captured LDR images when acquisition time is not a limiting factor.

The approach implemented in this study is similar to performing a Monte Carlo evaluation of noise propagation for HDR luminance imaging. For the uncorrelated sources of errors, the results obtained here could also be obtained by applying an analytical approach of the uncertainty propagation, following the principles of the GUM [20]. However, the simulation-based approach also enabled us to assess the impact of certain acquisition parameters for which correlations must be considered.

Finally, although many sources of error have been considered in our evaluation, it should be noted that the camera model proposed here represents an optically ideal camera, as no stray light effects were considered. Stray light (or lens glare), caused by unwanted scattering and reflections within the objective lens, as well as diffraction at the lens aperture, is often the dominant source of error affecting dark areas when the imaged scene presents high luminance contrasts. For a fixed scene geometry and lens aperture, the optical path of light rays within the objective lens remains the same regardless of the integration time. Therefore, considering stray light would not have provided additional insight into the optimal choice of the HDR merging algorithm. However, ILMD users should be fully aware of its significant impact on luminance measurement accuracy, as observed in preliminary studies based on experimental measurements [18]. We aim at further extending this work through dedicated experimental studies focusing on stray light characterization and its impact on luminance uncertainty. Once a realistic stray light model, applied as a convolution by a point spread function (see Figure 5), has been incorporated into HDR image formation, the proposed approach could first be confronted with experimental measurements and then applied to consider more realistic scenes, including geometrical information. This would allow extending this work beyond purely luminance measurement issues. In particular, coupling the radiometric model with disability glare or Human Visual System (HVS) models would enable the investigation of perceptual questions.

## Figures and Tables

**Table 1:** Varied parameters that characterize the camera properties. Parameters in bold are used to define a reference camera model.

Symbol [Unit]	Parameter	Tested Values
*εt*_int_ [ms]	Systematic error in the integration time	0, **0.002**, 0.005, 0.01
*K* [count·electrons^−1^]	Overall sensor gain [17]	No noise, **1**, 3, 10
*D*_offset_ [count]	Dark signal offset	0, **2**, 5, 10
*r*th [count·ms^−1^]	Rate of count generated due to thermal effects	0, **0.05**, 0.15, 0.3
(*a*, *b*)	Sensor flux non-linearity parameters (or residual non-linearity after correction)	Linear, **(0.03,2)**, (0.03,1), (0.05,1), (0.1,2), (0.1,1)
*σ*_read_ [count]	Standard deviation of readout noise	0, **3**, 7, 15
*Nbits* [bits]	Sensor bit depth	8, **12**, 16

**Table 2:** Varied parameters that characterize the acquisition of LDR images. Parameters in bold are used to define a reference camera model.

Symbol [Unit]	Parameter	Tested Values
*f*_t_	Integration time reduction factor	2, **4**, 8
*t*_min_ [ms]	Shortest integration time	**0.1**, 1, 10
*t*_max_ [ms]	Longest integration time	2000, **5000**, 8000

**Table 3:** Varied parameters that characterize the HDR merging algorithm. Parameters in bold are used to define a reference camera model.

Symbol [Unit]	Parameter	Tested Values
(*D*_min_, *D*_max_) [%]	Threshold applied to define the range of well-exposed pixels, as a percentage of the sensor’s dynamic range	(2%, 95%), **(5%,90%)**, (10%, 80%)

**Table 4:** Varied parameters that characterize the captured luminance scene. Parameters in bold are used to define a reference camera model.

Symbol [Unit]	Parameter	Tested Values
*σ*_source_ [%]	Standard deviation of the source temporal fluctuation, in percentage of its average value. The temporal periodicity Δ*t*_source_ = 10 µs is kept constant.	0%, **1%**, 2%, 5%

## Data Availability

The data and MATLAB codes supporting the conclusions of this article are made available by the authors on a repository at https://github.com/HiDyn-EURAMET-EPM-21NRM01/HDR_numerical-eval_HIDYN. (accessed on 13 January 2026) The MATLAB HDR algorithm codes for merging HDR images outside the evaluation framework are also available at https://github.com/HiDyn-EURAMET-EPM-21NRM01/HDRmerge_HYDIN (accessed on 13 January 2026).
